# Editorial: Immune response changes in alcohol and non-alcohol associated tissue injury

**DOI:** 10.3389/fimmu.2025.1559143

**Published:** 2025-01-31

**Authors:** Paul G. Thomes

**Affiliations:** Department of Anatomy, Physiology and Pharmacology, College of Veterinary Medicine, Auburn University, Auburn, AL, United States

**Keywords:** alcohol, immune response, tissue injury, therapeutic targets, treatment

The Research Topic, “*Immune Response Changes in Alcohol and Non-Alcohol Associated Tissue Injury”*, hosted by Frontiers in Immunology, brought together 10 articles. Six articles were related to alcohol-associated tissue injury, including two human studies and three rodent-based research articles. Non-alcohol-related contributions included liver stiffness in HBV-induced hepatitis, Paneth cell-targeting for radiation-induced gut injury and β-catenin’s role in hepatocellular carcinoma. A review article examined the impact of neutrophil extracellular traps (NETs) on NAFLD and potential therapeutic strategies. Immune responses are vital for mitigating damage and promoting tissue repair, but aberrant immune activation lead to chronic inflammation and organ dysfunction. This Research Topic highlights diverse contributions exploring immune dysregulation, offering insights into both alcohol- and non-alcohol-associated conditions.

The highlight of the Research Topic is two human studies on Alcohol Use Disorder (AUD). The first, by Vatsalya et al., investigated the gut-brain axis, focusing on alcohol withdrawal, withdrawal-associated depression, and cravings. The study showed that patients with significant withdrawal symptoms exhibited elevated gut permeability markers, such as lipopolysaccharide (LPS) and soluble CD14 (sCD14), alongside increased adiponectin and proinflammatory cytokines (e.g., IL-6, IL-8). Adiponectin was significantly associated with withdrawal severity, reflecting its role in inflammation and metabolic signaling. Depression scores correlated with gut dysfunction and cytokine responses, while cravings were linked to decreased leptin and elevated inflammatory cytokines (IL-1β, TNF-α). This study also highlights sex differences, with women displaying heightened immune dysregulation and severe AUD symptoms, suggesting therapeutic targeting of gut-brain axis mechanisms.

Building on this focus, the second human study, conducted by Sagaram et al., highlighted IL-22 and IL-17 as key cytokines in differentiating and understanding the progression of alcoholic hepatitis (AH). IL-22 levels were elevated in AH patients compared to those with AUD without liver injury, correlating with liver injury markers (AST, AST: ALT ratio) and lifetime drinking history. IL-22 demonstrated anti-inflammatory effects in non-severe AH subjects but became pro-inflammatory in subjects with severe AH, reflecting disease progression. IL-17 levels were highest with severe AH and correlated with MELD scores, indicating unresolved inflammation. These findings highlight IL-22 and IL-17 as key contributors to ALD progression, offering biomarkers and potential therapeutic targets for managing the disease.

Extending the insights from the human studies to preclinical models, Yue et al., explored IL-22’s protective mechanism in a preclinical model of AH. In mice, chronic alcohol exposure reduced intestinal IL-22 levels and impaired antimicrobial peptide (AMP) production and gut barrier integrity, leading to bacterial translocation and liver inflammation. IL-22 treatment however, effectively reversed these effects by restoring AMPs (e.g., Reg3, α-defensins), enhancing gut barrier proteins like ZO-1, and promoted the growth of beneficial microbiota such as Akkermansia spp. Additionally, IL-22 treatment improved liver histology, reduced lipid accumulation, and suppressed inflammatory cytokines via STAT3 activation in intestinal epithelial cells. These findings reinforce the potential of IL-22 as a therapeutic target for AH, demonstrating its role in gut-liver axis regulation and its relevance to human studies.

Further complementing these studies, Meena et al., investigated the role of TRPV6, a calcium-permeable ion channel, in stress and corticosterone-mediated exacerbation of alcohol-induced gut barrier dysfunction and systemic inflammation in mice. Chronic alcohol feeding disrupted intestinal tight and adherens junctions, increased gut permeability, and led to endotoxemia, systemic inflammation, and liver damage in wild-type mice. These effects were reversed in TRPV6-deficient mice. TRPV6 deficiency also prevented alcohol and stress-induced intestinal dysbiosis. These findings identify TRPV6 as a key mediator of gut-liver axis disruption under alcohol and stress conditions, presenting it as a potential therapeutic target.

Building molecular pathways in alcohol-induced gut-liver dysfunction, Walter et al. investigated the role of hepatocyte-specific mitogen-activated protein kinase phosphatase 1 (MKP1) in ALD. The study revealed that alcohol downregulated MKP1 expression, with females exhibiting greater susceptibility to liver injury. In MKP1-knockout mice, alcohol exposure led to severe liver damage, characterized by increased inflammation, endoplasmic reticulum stress (ER), and heightened c-Jun N-terminal kinase (JNK) activity, with these effects more pronounced in females. These findings emphasize MKP1’s protective role in mitigating alcohol-induced liver injury and its potential as a therapeutic target to address sex-specific risks in ALD.

Complementing these mechanistic insights, Aghara et al., reviewed the stress mechanisms driving the progression of ALD and evaluated the potential of nanoparticle-mediated therapies. The review identified oxidative stress, ER stress, and gut dysbiosis as key factors exacerbating ALD by promoting inflammation, mitochondrial dysfunction, and hepatic stellate cell activation. The review highlights that while early stages of ALD can be reversed with lifestyle changes, advanced stages require innovative treatments and Nanoparticles show promise in targeting ALD mechanisms, improving drug delivery, and enhancing bioavailability.

Transitioning from alcohol-related tissue injury, this Research Topic expands to non-alcohol-related liver and gastrointestinal conditions, highlighting mechanisms and therapeutic targets in fibrosis, viral infections, liver cancer, and gastrointestinal injury.

Bybee et al., investigated how increased liver stiffness contributes to the progression of hepatitis B virus (HBV) infection. Using liver fibrosis model, they reveal that elevated stiffness upregulates osteopontin (OPN), which suppresses interferon-stimulated genes (ISGs) and impairs antiviral immunity. This suppression promotes higher HBV RNA, DNA, and antigen levels, exacerbating liver fibrosis and inflammation. *In vitro* experiments confirmed that fibrotic conditions directly enhance HBV replication through OPN-mediated immune evasion, while *in vivo* data linked fibrosis severity to HBV progression. These findings highlight liver stiffness as a critical factor in HBV pathogenesis and a potential therapeutic target.

Nakagawa et al., examined how β-catenin stabilizes hypoxia-inducible factor 2 (HIF-2) through long non-coding RNAs (lncRNAs) in hepatocellular carcinoma (HCC). They found that β-catenin and NANOG activate the lncRNA EGLN3-AS1, which suppresses prolyl hydroxylase domain-containing protein 3 (PHD3), stabilizing HIF-2 and promoting tumor-initiating cells (TICs). This pathway supports tumor growth and resistance to immunotherapy. Targeting β-catenin with PRI-724 enhanced the efficacy of hepatitis B (HBIG) and C (HCIG) immunoglobulins, reducing TIC viability and tumor growth. The study highlights lncRNA pathways as potential therapeutic targets to improve immunotherapy for HCC.

Shukla et al., investigated gastrointestinal injury, specifically, the role of Paneth cells and human defensin 5 (HD5) in mitigating radiation-induced gut injury in mice. Their study found that total body irradiation in mice reduced Paneth cell α-defensin levels, leading to gut microbiota dysbiosis, increased intestinal permeability, endotoxemia, and systemic inflammation. Prophylactic HD5 supplementation prevented radiation-induced dysbiosis, restored tight and adherens junction integrity, and reduced inflammatory cytokines. Post-radiation HD5 treatment partially reversed these effects, including dysbiosis and barrier dysfunction. These findings highlight α-defensins role in gut immune regulation and suggest HD5 as a therapeutic agent for preventing and mitigating radiation-induced gastrointestinal injury in mice.

Concluding this section, Fa et al., reviewed the role of neutrophil extracellular traps (NETs) in the progression of nonalcoholic fatty liver disease (NAFLD). NETs, released by activated neutrophils, were shown to drive oxidant stress, inflammation, and immune cell recruitment, contributing to various stages of liver disease, including steatosis, steatohepatitis, fibrosis, and HCC. The review highlights NETs as potential therapeutic target and discusses interventions such as DNase-based treatments, antioxidants, and microbiota modulation to mitigate NET-driven liver injury and slow disease progression.

In conclusion, this Research Topic highlights diverse research on immune modulation in alcohol- and non-alcohol-associated tissue injury, providing new insights into disease progression and potential therapeutic targets for liver and gastrointestinal diseases.

